# Awareness of Hepatitis C Among the General Population of Riyadh, KSA, in 2023: A Cross-Sectional Study

**DOI:** 10.7759/cureus.51783

**Published:** 2024-01-07

**Authors:** Waqar A Farooqi, Lamees M AlGubran, Talal M Abukaram, Lama K Alharbi, Roaa A Alsanea, Ghuzlan A Zubaidi, Shahid A Alnassr, Tajah M Alaithan, Jenan M Alnamly, Qamar A Altriny

**Affiliations:** 1 College of Internal Medicine, AlMaarefa University, Riyadh, SAU; 2 College of Medicine, AlMaarefa University, Riyadh, SAU

**Keywords:** infection, viral, knowledge, hepatitis, liver diseases

## Abstract

Background

Hepatitis C, a highly contagious viral infection transmitted through blood-to-blood contact, poses a significant threat to public health owing to its potential to induce extensive liver damage, leading to the development of life-threatening conditions such as liver cirrhosis and hepatocellular carcinoma, commonly known as liver cancer.

Objective

This cross-sectional study aims to assess the level of awareness of hepatitis C among the general population in Riyadh, Saudi Arabia.

Methodology

The study was conducted in Riyadh, Saudi Arabia. A national cross-sectional survey was conducted using a predesigned self-administered validated electronic questionnaire. The questionnaire was distributed electronically to the general population from April to September 2023.

Results

Out of the 800 participants, most were females (472, 59%), and the largest age group was between 23 and 29 years old (305, 38.1%). Saudi Arabians constituted the highest proportion of participants (609, 76.1%), and most participants were single (437, 54.6%). In terms of occupational status, 288 (36%) worked in occupations not listed. The study found that 463 (57.9%) participants had a low level of awareness about the global prevalence of hepatitis C. Additionally, 489 (61.1%) were aware that hepatitis C can cause both acute and chronic hepatitis, and 441 (55.1%) knew that most infected patients may develop chronic hepatitis. A majority (484, 60.5%) were aware of the importance of washing with soap and water after an accidental needle stick injury. Most participants (574, 71.8%) were aware of the common modes of hepatitis C transmission, such as unsterilized instruments, needle stick injuries, and sharing contaminated needles during drug use. Furthermore, 548 (73%) were aware of the transmission risk through unscreened blood transfusion, and 561 (70.1%) were aware of the higher risk of infection for individuals with multiple sexual partners.

Conclusion

The study highlights a low level of awareness among participants regarding the treatment and prevention of hepatitis C. To address the low level of awareness regarding the treatment and prevention of hepatitis C, targeted strategies and interventions are needed. This includes the development and implementation of public awareness campaigns, creation of informative educational materials, specialized training programs for healthcare professionals, establishment of support groups and counseling services for individuals living with hepatitis C, and continued support for research efforts and knowledge dissemination. By increasing awareness about hepatitis C, its treatment, and prevention, we can improve outcomes for individuals affected by the disease and reduce its overall burden on communities.

## Introduction

Hepatitis C is a viral infection of the liver that can lead to severe liver damage and, in some cases, liver cancer [1]. The hepatitis C virus typically does not show noticeable symptoms until it progresses to more advanced stages [2]. The transmission of the hepatitis C virus occurs through direct contact with the blood of an infected individual [3]. As per the World Health Organization (WHO), around 71 million individuals globally are affected by chronic hepatitis C. In Saudi Arabia, the estimated prevalence of hepatitis C ranges from 0.4% to 2% [3-5].

Saudi Arabia, with a population of over 34 million, has improved its healthcare system to control and prevent hepatitis C [4]. The prevention of viral spread, particularly in the case of hepatitis, hinges upon the possession of comprehensive knowledge and the active promotion of awareness regarding these infections. This critical understanding not only empowers individuals to take the necessary precautions but also enables healthcare professionals to effectively diagnose, treat, and educate patients about the risks and preventive measures associated with these viruses [6-9].

Numerous comprehensive studies and a multitude of scholarly articles have consistently highlighted a disconcerting trend: the level of knowledge and awareness surrounding the hepatitis C virus is alarmingly low. Despite the availability of extensive information and resources, a significant portion of the population remains uninformed about the causes, transmission methods, symptoms, and potential consequences of this infectious disease. This lack of awareness not only hampers early detection and timely treatment but also perpetuates the stigma associated with hepatitis C, hindering efforts to combat its spread and improve public health outcomes. It is imperative that concerted efforts be made to bridge this knowledge gap through targeted educational campaigns, community outreach programs, and healthcare provider training, thereby empowering individuals with the necessary information to protect themselves and others from the perils of hepatitis C [10-14].

Surprisingly, even among nonspecialist physicians, there exists a concerning lack of understanding when it comes to hepatitis [15]. A significant proportion of individuals affected by hepatitis can be attributed to the widespread lack of awareness, particularly regarding the modes of transmission associated with this infectious disease. Numerous studies have consistently shown that a considerable number of people exhibit low levels of knowledge when it comes to how hepatitis is transmitted. This knowledge gap contributes to the continued prevalence of hepatitis, as individuals may unknowingly engage in behaviors that put them at risk of contracting or spreading the virus. Addressing this issue necessitates comprehensive educational campaigns and targeted interventions aimed at increasing awareness about the modes of transmission, thereby empowering individuals to make informed decisions and take necessary precautions to prevent the spread of hepatitis [16-19].

The findings of the research project were intended to assist policymakers, health professionals, and communities in Saudi Arabia in developing effective interventions to increase awareness among those at a higher risk of hepatitis C. The study aimed to identify critical areas requiring more education and awareness campaigns for the prevention and control of hepatitis C in the country, and the results were expected to be significant for estimating the prevalence and incidence rates of hepatitis C in the general population of Riyadh.

## Materials and methods

Study design

This study is a cross-sectional study exploring the knowledge and perception of the population of Saudi Arabia about hepatitis C.

Study area and population

The study was conducted in Riyadh, Saudi Arabia, which is the capital of Saudi Arabia and the largest city on the Arabian Peninsula.

Inclusion criteria

The adult population in Riyadh aged 18 years and above.

Exclusion criteria

People with mental disorders.

Sample size and technique

The sample size of 806 was determined based on the assumption of 50% awareness and 5% precision, multiplied by two for the design effect in a cross-sectional design, and with an additional 5% nonresponse rate. The study utilized cluster sampling, assuming equal population distribution among the five Riyadh clusters (south, east, west, north, and center of the city), with one shopping mall selected from each region. Participants were chosen through convenience sampling, and the study involved five trained data collectors who conducted face-to-face interviews with participants in various malls.

Data needs

The research employs a predesigned self-administered validated bilingual questionnaire, which has been adapted from a previous study and translated into Arabic. The questionnaire encompasses four main categories: demographic information, medical knowledge, identifying the causes, and follow-up and management.

Data analysis

Proportions and bivariate analysis were computed, and the appropriate significance test was employed to examine the data. Furthermore, a scoring system was developed to evaluate the participants' level of awareness and understanding regarding hepatitis C. Statistical significance was determined considering P values of 0.05 or less as significant.

Instruments and validation

The initial part of the survey gathered sociodemographic information such as gender, age, nationality, marital status, and occupational status. Subsequent sections comprised detailed questionnaires focusing on various aspects of hepatitis C, including medical knowledge, complications, transmission, screening, treatment, and prevention. Participants were asked to indicate their awareness by choosing between "I AM AWARE" and "I AM NOT AWARE" for each question. To assess their knowledge, we set a criterion based on their responses, considering those aware of more than 60% of the questions to have good knowledge and categorizing those unaware as having poor knowledge. It is worth noting that the questionnaire was conducted in Arabic, and we ensured the accuracy and completeness of the surveys before data entry. Part of the questionnaire was validated by EMCDDA [20], and the other part was through both content and face validity.

Ethical considerations

As per the ethical guidelines and regulations, this research study has obtained the necessary Institutional Review Board (IRB) approval, specifically IRB23-052, from AlMaarefa University in Riyadh, Saudi Arabia.

## Results

Table 1 displays the demographic characteristics of the 800 individuals who participated in the study. It reveals that the majority of the participants, comprising 472 (%=59) of the total, are women. The largest age group among the participants falls between 23 and 29 years old, comprising 305 (%=38.1) of the sample, while the smallest age group consists of individuals aged 30-39, accounting for 146 (%=18.3) of the participants. In terms of nationality, Saudi Arabians represent the highest proportion at 609 (%=76.1), while non-Saudis comprise 191 (%=23.9) of the participants. Regarding marital status, the majority of the participants are single, comprising 437 (%=54.6), while widows constitute the minority (N=12, %=1.5). The occupational status of the participants includes individuals working in business, engineering, and healthcare (physicians, nurses, pharmacists) as well as housewives and those employed in other occupations. The majority of participants, accounting for 288 (%=36), work in occupations other than those listed, while housewives make up the minority (N=75, %=9.4).

**Table 1:** The demographic information of the participants. N represents the frequency count of participants, while % represents the percentage that frequency accounts for out of the total data.

Parameter	Categories	N	%
Gender	Male	328	41.0
	Female	472	59.0
	Total	800	100.0
Age	18-22	172	21.5
	23-29	305	38.1
	30-39	146	18.3
	<40	177	22.1
	Total	800	100.0
Nationality	Saudi	609	76.1
	Non-Saudi	191	23.9
	Total	800	100.0
Marital Status	Single	437	54.6
	Married	299	37.4
	Divorced	52	6.5
	Widow	12	1.5
	Total	800	100.0
Occupational Status	Business	121	15.1
	Engineer	78	9.8
	Healthcare provider	238	29.8
	Housewife	75	9.4
	Other	288	36.0
	Total	800	100.0

Table 2 presents the responses to questions related to medical knowledge and complications associated with hepatitis C. The first question assessed the participants' awareness of the percentage of the world population infected with hepatitis C. The majority (N=463, %=57.9) demonstrated a low level of awareness, while the minority (N=337, %=42.1) were aware of this statistic. In terms of understanding that hepatitis C can cause both acute and chronic hepatitis, 489 (%=61.1) of the participants were aware, while 311 (%=38.9) were not. When asked if most infected patients may develop chronic hepatitis, the majority (N=441, %=55.1), displayed a high level of awareness, while 359 (%=44.9) were not aware. Additionally, 403 (%=50.4) of the participants were aware that some individuals can spontaneously clear the virus after infection and avoid developing a chronic infection, while the minority (N=397, %=49.6) were aware of this fact. Inquiring about the contraindication of pregnancy, 430 (%=53.8) of the participants were aware that it is not contraindicated. Regarding the signs and symptoms of hepatitis C, 456 (%=57) of the participants were aware that most patients remain asymptomatic for many years, while 344 (%=43) were not aware. Furthermore, when asked about the potential consequences of untreated hepatitis C, such as cirrhosis and liver carcinoma, 476 (%=59.5%) of the participants were aware, while 324 (%=40.5) were not. Finally, inquiring about the importance of washing with soap and water immediately after an accidental needle stick injury, 484 (%=60.5) of the participants were aware of this practice, while 316 (%=39.5) were not aware.

**Table 2:** Responses regarding medical knowledge and complications questions related to hepatitis C. N represents the frequency count of participants, while % represents the percentage that frequency accounts for out of the total data.

Parameter		N	%
As of 2022, 3% of the world population was infected with hep C (WHO).	o I AM AWARE	337	42.1
	o I AM NOT AWARE	463	57.9
Hepatitis C can cause both acute and chronic hepatitis.	o I AM AWARE	489	61.1
	o I AM NOT AWARE	311	38.9
Most patients who get infected with hepatitis C develop chronic hepatitis.	o I AM AWARE	441	55.1
	o I AM NOT AWARE	359	44.9
After infection, some people can spontaneously clear the virus from their body (about 30%) and do not develop chronic infection.	o I AM AWARE	403	50.4
	o I AM NOT AWARE	397	49.6
Pregnancy is not contraindicated in a woman with hepatitis C. It is the woman's choice.	o I AM AWARE	430	53.8
	o I AM NOT AWARE	370	46.3
Most patients with chronic hepatitis C remain asymptomatic for many years.	o I AM AWARE	456	57.0
	o I AM NOT AWARE	344	43.0
Untreated hepatitis C can lead to cirrhosis and liver carcinoma in some people.	o I AM AWARE	476	59.5
	o I AM NOT AWARE	324	40.5
In case of accidental needle stick injury, you should immediately wash with soap and water and report to the infection control department of your facility.	o I AM AWARE	484	60.5
	o I AM NOT AWARE	316	39.5

Table 3 presents the responses regarding awareness of transmission-related questions about hepatitis C. The participants were asked about common modes of transmission, such as the use of unsterilized dental and surgical instruments, needle stick injuries, and IV drug abuse with the sharing of contaminated needles. The majority (N=574, %=71.8) were aware of these modes of transmission, while 226 (%=28.2) were not. Inquiring about vertical transmission from mother to fetus, 464 (%=58) of the participants were aware that it can occur, while 336 (%=28.2) were not. When asked about the transmission of HCV through sharing needles for drug injection or tattooing, 588 (%=73.5) were aware, while 212 (%=26.5) were not aware. Regarding the transmission through the sharing of straws used for snorting drugs, 431 (%=53.9) were aware, while 369 (%=46.1) were not aware. The participants were also asked about the transmission through transfusion of unscreened blood or blood products, and 584 (%=73) were aware, while 216 (%=27) were not. Additionally, 561 (%=70.1) of the participants were aware that people with multiple sexual partners are at risk of getting infected, while 239 (%=29.9) were not aware. It was clarified that hepatitis C is not transmitted through food, water, saliva, breast milk, or aerosol, and 46 (%=58.3) of the participants were aware of this, while 334 (%=41.8) were not aware. Finally, when asked about casual contact like hugging or sharing food or drinks with an infected person, 500 (%=62.5) were aware that it is not transmitted, while 300 (%=37.5) were not aware. Overall, the findings indicate a high level of awareness regarding the transmission of hepatitis C.

**Table 3:** Responses regarding awareness of transmission questions related to hepatitis C. N represents the frequency count of participants, while % represents the percentage that frequency accounts for out of the total data.

Parameter	Aware or not	N	%
Common modes of transmission are the use of unsterilized dental and surgical instruments, needle stick injuries, and IV drug abuse with the sharing of contaminated needles.	o I AM AWARE	574	71.8
	o I AM NOT AWARE	226	28.2
Vertical transmission (mother to fetus) can occur, but it is low (about 2% chance).	o I AM AWARE	464	58.0
	o I AM NOT AWARE	336	28.2
HCV can be transmitted by sharing needles for drug injection or tattooing.	o I AM AWARE	588	73.5
	o I AM NOT AWARE	212	26.5
HCV can be transmitted by sharing of straws used for snorting drugs.	o I AM AWARE	431	53.9
	o I AM NOT AWARE	369	46.1
It can be transmitted by transfusion of unscreened blood/blood products.	o I AM AWARE	584	73.0
	o I AM NOT AWARE	216	27.0
People with multiple sexual partners are at risk of getting infected.	o I AM AWARE	561	70.1
	o I AM NOT AWARE	239	29.9
Hep C is not transmitted through food, water, saliva, breast milk, or aerosol.	o I AM AWARE	466	58.3
	o I AM NOT AWARE	334	41.8
It is not transmitted by casual contact like hugging or sharing food or drinks with an infected person.	o I AM AWARE	500	62.5
	o I AM NOT AWARE	300	37.5

Table 4 presents the responses regarding awareness of screening-related questions about hepatitis C. The participants were asked if checking for hepatitis C antibody (antiHCV) in the blood should be the first test for diagnosis. The findings show that 452 (%=56.5) of the participants had a high level of awareness, while 348 (%=43.5) had a low level of awareness. When asked about the next step, which is checking the virus in the blood by PCR to confirm its presence, the majority (N=413:%=51.6) were not aware, while 387 (%=48.4) were aware. The participants were also asked if the anti-HCV antibody could be a false negative in early infection. The majority (N=422, %=52.8) were not aware, while only 378 (%=47.3) were aware. Additionally, when asked if the anti-HCV antibody, which appears in the blood after infection, cannot eliminate the virus, 432 (%=54) were not aware, while 368 (%=46) were aware. Inquiring about the recommendation of routine testing for people in a high-risk category by the WHO, 446 (%=55.8) of the participants were aware, while 354 (%=44.3) were not aware. Furthermore, when asked if liver enzymes in the blood (AST, ALT) are often normal or minimally high even if the patient is infected, the majority (N=429, %=53.6) were not aware, while 371 (%=46.4) were aware. The participants were also asked if liver biopsy is not needed for diagnosis, and 422 (%=52.8) were not aware, while 378 (%=47.3) were aware. In summary, the findings indicate varying levels of awareness among the participants regarding screening-related aspects of hepatitis C.

**Table 4:** Responses regarding awareness of screening questions related to hepatitis C. N represents the frequency count of participants, while % represents the percentage that frequency accounts for out of the total data.

Parameter	Aware or not	N	%
Checking for hepatitis C antibody (antiHCV) in the blood should be the first test for diagnosis.	o I AM AWARE	452	56.5
	o I AM NOT AWARE	348	43.5
The next step is to check the virus in the blood by PCR to confirm its presence.	o I AM AWARE	387	48.4
	o I AM NOT AWARE	413	51.6
Anti-HCV antibodies can be false negative in early infection.	o I AM AWARE	378	47.3
	o I AM NOT AWARE	422	52.8
The anti-HCV antibody, which appears in the blood after infection, cannot eliminate the virus.	o I AM AWARE	368	46.0
	o I AM NOT AWARE	432	54.0
WHO recommends routine testing of people in a high-risk category.	o I AM AWARE	446	55.8
	o I AM NOT AWARE	354	44.3
Liver enzymes in the blood (AST, ALT) are often normal or minimally high even if the patient is infected.	o I AM AWARE	371	46.4
	o I AM NOT AWARE	429	53.6
A liver biopsy is not needed for the diagnosis.	o I AM AWARE	378	47.3
	o I AM NOT AWARE	422	52.8

Table 5 presents the responses regarding the awareness of treatment and prevention-related questions about hepatitis C. The participants were asked about newer agents called "direct-acting antivirals" (DAA), which are oral pills that greatly improve cure rates and result in most people being cured. The majority (N=479, %=59.9) were not aware of this, while 321 (%=40.1) were aware. When asked about the WHO's recommendation for the use of direct-acting antivirals in all patients aged three years and above, 485 (%=60.6) were not aware, while 315 (%=39.4) were aware. Inquiring about the necessity of combination therapy with two or more drugs to avoid the development of resistance to the virus, 435 (%=54.4) were not aware, while 365 (%=45.6) were aware. The participants were also asked about the duration of treatment, with 440 (%=55) not being aware that it is typically given for about three months, while 360 (%=45) were aware. It was clarified that, even after treatment, a person can get reinfected if exposed to the risk factors, and 422 (%=52.8) of the participants were aware of this, while 378 (%=47.3) were not. Additionally, 420 (%=52.5) of the participants were aware that there is no vaccine for hepatitis C, while 380 (%=47.5) were not aware. Finally, when asked about the availability of commercially available immune globulins against the hepatitis C virus, 490 (%=61.3) were not aware, while 310 (%=38.8) were aware. In summary, the findings indicate a low level of awareness among the participants regarding treatment and prevention-related aspects of hepatitis C.

**Table 5:** Responses regarding the awareness of treatment and prevention questions related to hepatitis C. N represents the frequency count of participants, while % represents the percentage that frequency accounts for out of the total data.

Parameter	Aware or not	N	%
Newer agents called “direct-acting antivirals” (DAA) which are oral pills, have greatly improved cure rates, and most people now get cured.	o I AM AWARE	321	40.1
	o I AM NOT AWARE	479	59.9
WHO recommends the use of direct-acting antivirals in all patients three years of age and above.	o I AM AWARE	315	39.4
	o I AM NOT AWARE	485	60.6
Treatment should always be a combination of two or more drugs to avoid the development of resistance to the virus.	o I AM AWARE	365	45.6
	o I AM NOT AWARE	435	54.4
Treatment is given for about three months.	o I AM AWARE	360	45.0
	o I AM NOT AWARE	440	55.0
Even after treatment, a person can get re-infected if he/she is exposed to the risk factors.	o I AM AWARE	422	52.8
	o I AM NOT AWARE	378	47.3
There is no vaccine for hepatitis C.	o I AM AWARE	420	52.5
	o I AM NOT AWARE	380	47.5
There are no commercially available immune globulins against the hepatitis C virus.	o I AM AWARE	310	38.8
	o I AM NOT AWARE	490	61.3

Table 6 presents the relationship between knowledge of hepatitis C and the demographic information of the participants. Upon analyzing this awareness concerning various sociodemographic factors, we discovered statistically significant associations with age, marital status, and occupational status. However, no statistically significant relationship was observed with gender and nationality. Regarding age, participants above the age of 40 demonstrated a higher level of knowledge (N=89, %=50.3). In terms of marital status, singles exhibited a higher awareness of hepatitis C (N=197, %=45.1). When considering occupational status, individuals working in the healthcare sector displayed the highest level of awareness among all participants (N=165, %=69.3). Overall, the findings indicate that the majority of participants have a low level of awareness regarding hepatitis C, with significant variations observed based on age, marital status, and occupational status.

**Table 6:** The relationship between knowledge of hepatitis C and demographic information of the patients. Age (p=0.010): The P value for age indicates that it has a significant relationship. Marital Status (p=0.009): The P value shows that marital status is significantly related. Occupational Status (p=0.001): Occupational status has an even stronger significant relationship based on its lower P value.

		Knowledge			
Parameter		Good	Poor	Total	P value
Gender	Male	123 (37.5)	205 (62.5)	328 (41.0)	0.068
	Female	208 (44.1)	264 (55.9)	472 (59.0)	
	Total	331 (41.4)	469 (58.6)	800 (100)	
Age	18-22	67 (39.0)	105 (61.0)	172 (21.5)	0.010
	23-29	128 (42.0)	177 (58.0)	305 (38.1)	
	30-39	47 (32.2)	99 (67.8)	146 (18.3)	
	<40	89 (50.3)	88 (49.7)	177 (22.1)	
	Total	331 (41.4)	469 (58.6)	800 (100)	
Nationality	Saudi	251 (41.2)	358 (58.8)	609 (76.1)	0.933
	Non-Saudi	80 (41.9)	111 (58.1)	191 (23.9)	
	Total:	331 (41.4)	469 (58.6)	800 (100)	
Marital Status	Single	197 (45.1)	240 (54.9)	437 (54.6)	0.009
	Married	119 (39.8)	180 (60.2)	299 (37.4)	
	Divorced	13 (25.0)	39 (75.0)	52 (6.5)	
	Widow	2 (16.7)	10 (83.3)	12 (1.5)	
	Total:	331 (41.4)	469 (58.6)	800 (100)	
Occupational Status	Business	31 (25.6)	90 (74.4)	121 (15.1)	0.001
	Engineer	11 (14.1)	67 (85.9)	78 (9.7)	
	Healthcare provider	165 (69.3)	73 (30.7)	238 (29.8)	
	Housewife	16 (21.3)	59 (78.7)	75 (9.4)	
	Other	108 (37.5)	180 (62.5)	288 (36.0)	
	Total	311 (41.4)	469 (58.6)	800 (100)	

## Discussion

In this study, our primary objective was to comprehensively assess the level of awareness regarding hepatitis C among a diverse sample of 800 participants. Our findings revealed a notable variation in the level of awareness among the participants when it came to different aspects of hepatitis C. When evaluating the potential complications associated with hepatitis C, it was observed that the majority of participants held the belief that this viral infection can lead to the development of other serious diseases, such as liver cirrhosis and cancer. These findings are consistent with a study conducted by Alzahrani et al. in 2022, which reported similar results regarding the participants' awareness of the potential complications of hepatitis C [4].

Moving on to the assessment of the participants' knowledge regarding the modes of transmission, we specifically inquired about the possibility of transmission through blood, sharing needles, and sexual intercourse. Encouragingly, the majority of participants demonstrated a high level of awareness regarding these transmission routes. These findings align with a study conducted by Behzad et al. in 2019, which reported a high level of awareness among participants regarding the various modes of transmission for hepatitis C [6]. 

However, when it comes to the awareness of the available treatment options for hepatitis C, our findings indicated that the majority of participants exhibited a low level of awareness. This finding is consistent with many studies, which reported that most participants had an extremely low level of awareness regarding the treatment of hepatitis C [21-23].

Regarding screening, it is crucial to highlight that our study is in line with numerous other studies conducted in this field. These studies have consistently revealed a significant lack of awareness among many individuals regarding the importance and benefits of screening [24-26]. Screening plays a vital role in detecting diseases or conditions at an early stage when they may be more treatable or manageable. It involves the use of various tests or examinations to identify potential health issues before symptoms become apparent. However, despite its potential to save lives and improve health outcomes, many people remain unaware of its significance. The lack of awareness surrounding screening can have serious consequences. Without proper knowledge, individuals may not understand the importance of regular screenings or the potential risks associated with not undergoing them.

This lack of awareness can lead to delayed diagnoses, missed opportunities for early intervention, and ultimately, poorer health outcomes. Based on these significant findings, it is evident that there is a pressing need for increased awareness and education regarding the available treatment options for hepatitis C. Healthcare providers and public health organizations should prioritize the dissemination of accurate and up-to-date information about the various treatment modalities and their effectiveness. This can be achieved through targeted awareness campaigns, the development of informative educational materials, and the implementation of comprehensive healthcare provider training programs. By improving awareness and knowledge about hepatitis C treatment, we can enhance early detection, facilitate timely access to appropriate care, and ultimately improve patient outcomes.

The limitations of the study include potential selection bias, as the research is limited to the general population of Riyadh, KSA, in 2023. The findings may not be generalizable to other regions or periods. Additionally, the study relies on self-reported awareness of hepatitis C, which may be subject to recall bias or social desirability bias. The cross-sectional design limits the ability to establish causality or determine the temporal relationship between awareness and other variables. Furthermore, the study does not explore potential barriers to accessing healthcare or obtaining accurate information about hepatitis C. Therefore, the results should be interpreted within the context of these limitations, and further research is needed to validate and expand upon the findings.

## Conclusions

In conclusion, the findings indicate that there is a varying level of awareness among individuals regarding hepatitis C, particularly in terms of its treatment and prevention. While there is a relatively high level of awareness regarding the transmission routes and potential complications of hepatitis C, there is a significant lack of awareness regarding available treatment options. To address this, it is crucial to implement targeted strategies and interventions. These may include conducting public awareness campaigns, developing informative educational materials that provide accurate information about hepatitis C, its prevention, and available treatment options, and promoting continued research and knowledge dissemination. By doing so, we can work towards increasing awareness about hepatitis C, its treatment, and prevention, ultimately improving the outcomes for individuals affected by the disease.

## References

[1] Almansour AH, Darwish MA, Abdel Wahab MM (2017). Hepatitis C infection awareness among fourth year medical students at University of Dammam. J Family Community Med.

[2] Altraif I (2018). Can hepatitis C virus be eliminated by 2030? Saudi Arabia as an example. Saudi Med J.

[3] Ali E, Saleh IH, Eltahir MA (2019). Knowledge and awareness toward hepatitis C Infection among dental students in Qassim University, Saudi Arabia. J Dent Sci.

[4] Alzahrani MS, Ayn Aldeen A, Almalki RS (2023). Knowledge of and testing rate for hepatitis C infection among the general public of Saudi Arabia: a cross-sectional study. Int J Environ Res Public Health.

[5] Metwally AM, Elmosalami DM, Elhariri H (2021). Accelerating hepatitis C virus elimination in Egypt by 2030: a national survey of communication for behavioral development as a modelling study. PLoS One.

[6] Dehghani B, Dehghani A, Sarvari J (2020). Knowledge and awareness regarding hepatitis B, hepatitis C, and human immunodeficiency viruses among college students: a report from Iran. Int Q Community Health Educ.

[7] Naveed S, Qamar F, Zainab S, Sarwar G (2014). A survey study on awareness of hepatitis-C in different groups. World J Pharm Res.

[8] Gul F, Savul S, Aamir R, Zehra T, Mujtaba H, Sadiq F (2022). Knowledge and awareness of hepatitis B, hepatitis C, and HIV among pregnant women in Pakistan. J Infect Dev Ctries.

[9] Samuel ST, Martinez AD, Chen Y, Markatou M, Talal AH (2018). Hepatitis C virus knowledge improves hepatitis C virus screening practices among primary care physicians. World J Hepatol.

[10] Eleje GU, Rabiu A, Mbachu II (2021). Awareness and prevalence of hepatitis C virus infection among pregnant women in Nigeria: A national pilot cross-sectional study. Womens Health (Lond).

[11] Mallick N, Anjum F, Leghari QA, Bashir L, Naz S (2018). Awareness of hepatitis C in Karachi, Pakistan. World J Pharm Pharm Sci.

[12] Kim KA, Lee JS (2020). Prevalence, awareness, and treatment of hepatitis C virus infection in South Korea: evidence from the Korea National Health and Nutrition Examination Survey. Gut Liver.

[13] Choi GH, Jang ES, Kim JW, Jeong SH (2020). A survey of the knowledge of and testing rate for hepatitis C in the general population in South Korea. Gut Liver.

[14] Sultan NY, YacoobMayet A, Alaqeel SA, Al-Omar HA (2018). Assessing the level of knowledge and available sources of information about hepatitis C infection among HCV-infected Egyptians. BMC Public Health.

[15] Feng B, Zhang J, Wei L (2011). Inadequate awareness of hepatitis C among nonspecialist physicians in China. Adv Med Educ Pract.

[16] Rashiti-Bytyci A, Ramadani N, Rashiti P (2022). Hepatitis C in several risk groups of Kosovo. J Infect Dev Ctries.

[17] Wu E, Chen X, Guan Z (2015). A comparative study of patients' knowledge about hepatitis C in the United States and in urban and rural China. Hepatol Int.

[18] Nawaz K, Hussain M, Majeed I, Afzal M, Gilani SA (2018). Knowledge, attitude and practices regarding hepatitis C prevention among people of rural community, Lahore. Int J of Soc Sci Manag.

[19] Vermunt J, Fraser M, Herbison P, Wiles A, Schlup M, Schultz M (2015). Prevalence and knowledge of hepatitis C in a middle-aged population, Dunedin, New Zealand. World J Gastroenterol.

[20] (2024). Knowledge questionnaire on viral hepatitis for drug service staff. https://www.emcdda.europa.eu/drugs-library/knowledge-questionnaire-hepatitis-drug-service-staff_en.

[21] Garg R, Aggarwal S, Kaur S, Bansal P, Bhatia A (2015). Awareness and attitude appraisal toward hepatitis-C among North West population of India-a cross sectional study. BJMMR.

[22] Setia S, Gambhir R, Kapoor V, Jindal G, Garg S, Setia S (2013). Attitudes and awareness regarding hepatitis B and hepatitis C amongst health-care workers of a tertiary hospital in India. Ann Med Health Sci Res.

[23] Jamil MS, Ali H, Shaheen R, Basit A (2010). Prevalence, knowledge and awareness of hepatitis C among residents of three Union Councils in Mansehra. J Ayub Med Coll Abbottabad.

[24] Gok Sargin Z (2022). Awareness of chronic hepatitis C in the Western Black Sea Region. Eur Rev Med Pharmacol Sci.

[25] Valerio H, Conway A, Alavi M (2022). Awareness of hepatitis C virus infection status among people who inject drugs in a setting of universal direct-acting antiviral therapy: the ETHOS Engage study. Int J Drug Policy.

[26] Ju FB, Dumpe ML, Lane GB, Shanks LC (2020). Awareness, compliance, and barriers of 1-time hepatitis C screening in hospital-based providers. J Nurse Pract.

